# Environmental Regulation, Corporate Economic Performance and Spatial Technology Spillover: Evidence from China’s Heavily Polluting Listed Corporations

**DOI:** 10.3390/ijerph19031131

**Published:** 2022-01-20

**Authors:** Xuesong Gu, Xiaoran An, Andong Liu

**Affiliations:** School of Economics and Management, Beijing Forestry University, Beijing 100083, China; guxuesong@bjfu.edu.cn (X.G.); lad_1997@163.com (A.L.)

**Keywords:** environmental regulation, technology spillover, Porter hypothesis, corporate profitability, spatial econometrics

## Abstract

The relationship between environmental regulation, technology spillover, and economic performance has been the subject of intense scholarly debate in environmental economics for many years. The famous Porter hypothesis states that environmental regulation promotes both the economic performance and the environmental performance of corporations. However, the existing literature has paid relatively little attention to micro-level research and spatial spillover effects. This article endeavors to fill this gap by an empirical analysis of a sample of 900 of China’s heavily polluting listed corporations for the period of 2013–2016. By utilizing spatial econometric methods to measure spatial direct and indirect effects and decomposing total factor productivity change into technical change, pure efficiency change, and scale efficiency change, we find that environmental regulation promotes corporate total factor productivity but widens the disparity between profitable and unprofitable corporations. Our results also suggest that the direct and indirect effects of environmental regulation and corporate profitability on promoting total factor productivity rely heavily on the efficiency changes, while the contribution of the key component, technical change, is insignificant.

## 1. Introduction

China has undergone rapid economic development for many years, but it also suffers from severe environmental pollution. According to the 2016 China Environmental Status Bulletin released by the Ministry of Environmental Protection of the People’s Republic of China [1], among the 338 major cities in China, 84 cities achieved environmental air quality standards, accounting for 24.9% of all the cities, whereas 254 cities failed to meet the standards, accounting for 75.1%. The average proportion of good-quality days in these cities was 78.8%, while the average ratio of above-standard days was 21.2%. Precipitation monitoring was carried out in 474 cities (districts and counties). The proportion of cities with acid rain accounted for 19.8%, and the average frequency of the acid rain was 12.7%. In addition, among the 1940 evaluation sites of surface water in the country, approximately one third were classified into the last three grades of a six-grade assessment system. Among the 6124 groundwater-quality monitoring sites, more than 60% were the 4th and 5th grades in a five-grade system. Environmental degradation has become a notable problem facing China.

The Chinese economy has transformed from a stage of rapid growth to a stage of high-quality development. Improving environmental quality and developing a green economy are the keys to improving the quality of China’s economy. Therefore, environmental regulation is necessary to optimize China’s economic development mechanism. However, enhancing environmental regulation requires high costs, including financial pressure for the governments, regulatory costs for the regulatory authorities, and tax burdens for corporations, among others. On the other hand, heavily polluting corporations may need to abandon existing production models, update equipment, and invest in research and development activities to make them comply with environmental regulatory requirements, which means high explicit costs for those corporations that may harm profitability.

Is economic development incompatible with the improvement of environmental quality? Numerous scholars have done a lot of investigation in this field. The conclusions are presented in the literature review. Most studies adopt macro-level data, such as country-level, sector-level, and industry-level data, because collecting micro-level data, i.e., firm-level, is relatively difficult. Macro-level data contain more information, but micro-level data are more targeted and specific. Corporation-level data can thus be of great significance in terms of examining how corporations respond to environmental regulation because corporations are the recipients of environmental regulation, and their reactions are the basis for policymakers to adjust their environmental policies to achieve certain goals. Moreover, the environmental regulation measurement also has to match the data scale. With respect to this, the utilization of corporation-level single indices outnumbers that of the multidimensional indices. However, the latter can be a better approach because these indices are comprehensive and can more accurately measure how much environmental regulation burdens each corporation. Currently, there is only a very small proportion of the studies that possess both of the features above, namely the adoption of micro-level data and the construction of environmental regulation via multidimensional indices.

In addition, some researchers in this field have also considered spatial correlation, but in a more general way. Environmental regulation in one province can be influenced by that of other provinces because local governments are more or less involved in competition against each other or are influenced by the overall strategy of the country, which can cause them to make adjustments in their development strategies accordingly, including in environmental policymaking. For example, in order to clean up the capital’s environmental problems, the Chinese Government has introduced policies to limit production capacity in the provinces surrounding Beijing in order to improve the capital’s environmental conditions. In addition, China has introduced regional coordinated development policies, such as the Beijing-Tianjin-Hebei integration and the Yangtze River Delta integration, which enhance the regional relevance of the environmental regulations and other related policies and lead to more obvious spatial spillover effects. Similarly, competition among corporations is more obvious, leading to the spatial interaction of corporations in their technology spillovers and economic performances. As a result, it is of great value to study the spatial interaction of environmental regulation.

The greatest difference between this article and the previous literature is that our research simultaneously combines the three features mentioned above, namely the elaboration of the environmental regulation variable via multidimensional indices, the adoption of micro-level data, and the use of spatial econometric methods. 

To investigate the mechanism of how each component of total factor productivity (TFP) affects the overall TFP, this study decomposes TFP through the DEA-Malmquist index and conducts empirical analyses via the instrumental-variables two-stage least-squares (IV-2SLS) regression model and the spatial Durbin model, which can measure direct and indirect effects. Using the panel data of 900 of China’s heavily polluting listed corporations from 2013 to 2016, we discover that environmental regulation has a positive direct effect and a negative indirect effect on TFP; most of these effects are due to pure efficiency change and scale efficiency change. Corporate profitability exerts negative direct and indirect effects on TFP; most of these effects are due to pure efficiency change and scale efficiency change. Moreover, these effects may vary between profitable and unprofitable corporations.

### 1.1. The Present Study

Scholars have already been making contributions to studies on the relationship between environmental regulation, technological improvement, and economic performance for many years. Michael Porter [2] proposed the famous Porter hypothesis, which states that environmental regulation encourages corporations to innovate, and as a result, these corporations tend to gain a win-win situation in terms of economic performance and environmental performance. The majority of scholars agree with the Porter hypothesis [3,4,5,6]. However, some hold the view that environmental regulation restrains technological improvement [7,8,9]. There are also some authors arguing that the effects of environmental regulation on technological improvement are significant only under particular conditions [10,11,12]. However, the mechanism of how environmental regulation impacts technological improvement has not been fully researched.

When it comes to the spatial spillover effects of environmental regulation, competition between local governments gives local environmental regulation policies the power to impact the environmental regulation policymakers in other regions. Likewise, technological improvement in one corporation can easily affect other corporations due to the spillover effect of technologies. With respect to financial performance, the influences are even stronger in an environment with fierce competition because corporations are forced to react to the performances of their rivals.

Many studies diverge on how they choose sample data for research on the above issue. Some investigations are based on macro-level data [13,14,15]. However, some investigations chose to set their data at the micro-level [16,17,18]. Regardless of the level of data, the main problem is the number of industries in the sample, which can affect the generality of the conclusions. When the data level is taken into consideration, it can easily be found that for the macro-level data including more industries in the dataset is much easier than for the micro-level data due to their different availabilities.

A major difference between those studies in this field is how a proxy was chosen for the environmental regulation. Brunel and Levinson [19] thoroughly explained how difficult it is to construct an ideal variable to measure environmental regulation. Many scholars have established multidimensional indices to measure environmental regulation, both at the macro-level and the micro-level [20,21]. Those approaches share one feature, which is the incorporation of as many types of indices as possible in the variable. In contrast, single indices, such as pollution abatement and control expenditures [22], are not as comprehensive as the multidimensional indices. 

There is limited literature at a spatial level which can help us to investigate our subject matter spatially as the stringency of the environmental regulation in a certain area may influence that in other areas. The spatial econometric method was not introduced into the relevant research for the first time until this century, by Maddison [23], who tried to investigate environmental Kuznets curves spatially. Since then, little attention has been paid to introducing spatial econometric methods into related research. The application of spatial methodology requires specific data; so, at the micro-level, it is almost impossible to collect the ideal data to carry out this estimation. Feng and Chen [24] used provincial industrial panel data to research environmental regulation, green innovation, and industrial green development, which are close to the subject matter, but their data are at the macro-level.

In summary, the present study shows insufficient consideration in terms of sample size and environmental regulation measurement, as well as technological space spillover. The most important thing is that the application of spatial econometric methods has not yet been fully popularized. In a few studies involving this aspect, the authors prefer to use macro-level data. 

Compared with the existing research, we have made progress mainly in the aspect of the measurement of environmental regulation and the spatial spillover effects of the technology. This article represents the first application of the spatial econometric method to explore the nexus between environmental regulation, corporate profitability, and TFP in the micro-level field. First, we employ the quasi-environmental tax, a multidimensional index combining many administrative charges and taxes related to the environment, instead of a single index that has been used in many previous studies, to construct a more comprehensive environmental regulation index that may provide more convincing conclusions [25]. Second, to correspond with how we construct this variable, corporation-level panel data containing multiple industries are utilized; these include the data from all the available heavily polluting listed corporations in China. These data contribute more insights into this subject matter from the perspective of the micro-level that may help lead to a better understanding of how the mechanism of environmental regulation works. Third, we also introduce a spatial econometric method into our research to examine the technology spillover effects resulting from environmental regulations that were ignored previously but are of great value.

### 1.2. Theoretical Hypotheses

The effects of environmental regulation on the efficiency of corporations, especially heavily polluting corporations, have not yet been concluded. Zhou et al. [26] proposed that environmental regulation has significantly improved the profitability of heavily polluting companies through the consolidation of the enterprises’ cost management and the elimination of small firms with high compliance costs. Mbanyele and Wang [27] provided evidence showing that environmental regulation substantially promotes innovation productivity and that the impact is more pronounced for pollution-intensive industries. However, Nie et al. [28] believed that no evidence had been found in support of the idea that environmental regulation could stimulate the regional Porter effect.

Theoretically, environmental regulation puts forward higher requirements for the production activities of corporations, especially for the heavily polluting ones, which are the first-line targets of environmental governance. The reasons are as follows: firstly, environmental regulation has lower supervision intensity for non-heavily polluting corporations, whose effects are not as obvious as those of the heavily polluting corporations. In addition, non-heavily polluting corporations have fewer cost burdens in paying quasi-environmental taxes; so, they have no reasons to take measures which have the risk of increasing costs and destroying efficiency to change the status quo in order to reduce environmental pollution in the production process. In summary, it is less likely that environmental regulation spillover will penetrate into the non-heavily polluting corporations. As a result, compared with the non-heavily polluting corporations, the heavily polluting ones may achieve a greater degree of technical efficiency improvement through environmental regulation. Under such circumstances, heavily polluting corporations are forced to improve technology and innovate production methods to meet regulatory requirements. At the same time, the improvement of corporate technical efficiency will make the operating level and profitability more promising and ultimately boost the corporations’ overall efficiency.

Additionally, compared to the more profitable corporations, the unprofitable corporations are in more difficult situations under strict environmental regulation, which makes them bear more losses. Instead of suffering from continuous costs due to high fines or frequent restrictions, technical improvements should be made without interruption. Although it causes large expenses in the initial stage of research and development, the services of the new technology and equipment can not only eliminate environmental burdens but can also enhance operating efficiency. The above two reasons both prompt corporations to choose to improve technical efficiency.

According to the above literature analysis and theoretical analysis, we propose the following hypotheses:

**Hypothesis** **1** **(H1).** *Environmental regulation has positive effects on the heavily polluting corporations’ efficiency.*

**Hypothesis** **2** **(H2).** *Environmental regulation improves the efficiency of unprofitable corporations compared with profitable ones.*

The spatial interaction of environmental regulation is also a noteworthy topic. Recently, a few researchers have begun to consider it and to try to verify it quantitatively at the macro-level. The empirical results of Wu et al. [29] indicated that the strategic interaction of environmental regulation is evident among central and western cities in China. Peng et al. [30] concluded that in the face of strict environmental regulations, the geographical proximity accelerates the diffusion of green knowledge and technology. Liu et al. [31] found that environmental regulations have obvious direct and indirect effects on the impact of high-quality economic development in the central and eastern regions.

Suppose that the regional division is based on the provinces. When environmental regulation is implemented in one province, the technical efficiency and operating performance of the corporations over there will be improved. Due to fierce competition, the corporations in the other provinces will introduce the advanced technology to make their own performance better. On the other hand, the governments will also compete for environmental quality. When a province has made an achievement through the environmental regulation, others will implement similar measures. Therefore, the technical efficiency of the corporations located in the other provinces will increase accordingly.

Based on the above analysis, we propose the third hypothesis:

**Hypothesis** **3** **(H3).** *Environmental regulation leads to the spatial interaction of corporations in their technology spillovers.*

## 2. Materials and Methods

### 2.1. Methodology

#### 2.1.1. Calculation and Decomposition of TFP

With respect to how environmental regulation affects TFP, decomposing it into several components is a common practice. Most studies use the three decomposed components from TFP to illustrate how much each contributes to the growth rate of TFP [32,33]. In the following, we briefly introduce the method used to compute and decompose TFP.

- DEA model

This study intends to calculate the TFP of 900 corporations for the period of 2013–2016, referring to the DEA-Malmquist index methodology used by Fare et al. [34]. Initially, we regard these corporations as decision-making units (DMUs). Then, we assume that during every period t, the i-th corporation inputs n types of production elements $x_{i,n}^{t}$ (*n* = 2), which are the labor input $x_{i,1}^{t}$ and the capital input $x_{i,2}^{t}$. The output element $y_{i}^{t}$ is the business income. Under the condition of constant returns to scale (C) and stringently disposable input elements (S), the potential technical frontier can be defined as:

(1) $$L_{t}(y^{t}\left|C,\;S\right)=\{\left(x_{1}^{t},x_{2}^{t}\right)|y_{i}^{t}\leq \overset{900}{\underset{i=1}{\sum }}z_{i}^{t}y_{i}^{t};x_{i,n}^{t}\geq \overset{900}{\underset{i=1}{\sum }}z_{i}^{t}x_{i,n}^{t};z_{i}^{t}\geq 0\}$$

where $z_{i}^{t}$ denotes the weight of the DMUs when evaluating production efficiency. The efficiency based on the input of each DMU is presented as follows:

(2) $$\{\begin{array}{c} \begin{array}{c} F_{i}^{t}(x^{t},\;y^{t}{|C,\;S)=min\theta }_{i}\\ s.t.y_{i}^{t}\leq \overset{900}{\underset{i=1}{\sum }}z_{i}^{t}y_{i}^{t} \end{array}\\ \theta_{i}x_{i,n}^{t}\geq \overset{900}{\underset{i=1}{\sum }}z_{i}^{t}x_{i,n}^{t}\\ z_{i}^{t}\geq 0 \end{array}$$

where weight $z_{i}^{t}$ and $\theta_{i}$ are in the interval [0, 1], and the objective function value $F_{i}^{t}(x^{t},\;y^{t}\left|C,\;S\right)$ represents the distance of the DMU shifting from the technical frontier, which is a set of minimum inputs under a given output. When this value equals 1, the DMU is situated on the level of the technical frontier, where the production efficiency reaches the maximum. Taking the reciprocal of $F_{i}^{t}(x^{t},\;y^{t}\left|C,\;S\right)$, we obtain the input distance function $D_{i}^{t}(x^{t},\;y^{t})$ under the technical frontier level $L_{t}(y^{t}\left|C,\;S\right)$. Similarly, the other three distance functions can be obtained: $D_{i}^{t}(x^{t+1},\;y^{t+1})$, $D_{i}^{t+1}(x^{t},\;y^{t})$, and $D_{i}^{t+1}(x^{t+1},\;y^{t+1})$. Under the technical condition of period t, according to the methodology used by Caves et al. [35], we build the Malmquist index of the change of TFP from period *t* to period *t* + 1:

(3) $$M_{i}^{t}=\frac{D_{i}^{t}(x^{t+1},\;y^{t+1})}{D_{i}^{t}(x^{t},\;y^{t})}$$

where the distance function is defined as the reciprocal of the “maximum” proportional expansion of the output vector $y^{t}$, given the inputs $x^{t}$. In addition, it is the reciprocal of Farrell’s measure of output technical efficiency, which calculates “how far” an observation is from the frontier of technology. It completely characterizes the technology. In particular, note that $D_{i}^{t}(x^{t},\;y^{t})=1$ if and only if $\left(x^{t},\;y^{t}\right)$ is on the boundary or frontier of the technology, which occurs when the production is technically efficient. In addition, $M_{i}^{t}$ is the ratio of the reciprocal of efficiency at period *t* + 1 and period *t*, which represent the change of TFP.

In the same way, the Malmquist index of the change in TFP from period t to period *t* + 1, under the technical condition of period *t* + 1, can be presented as:

(4) $$M_{i}^{t+1}=\frac{D_{i}^{t+1}(x^{t+1},\;y^{t+1})}{D_{i}^{t+1}(x^{t},\;y^{t})}$$

To avoid the biased error resulting from the arbitrariness of period choice, we take the geometric mean of the two Malmquist indices, $M_{i}^{t}$ and $M_{i}^{t+1}$. Eventually, we acquire the formula measuring the change in TFP from period t to period *t* + 1:

(5) $$TFP_{i,t+1}\;=\;M_{i}(x^{t+1},\;y^{t+1};\;x^{t},\;y^{t})=[\frac{D_{i}^{t}(x^{t+1},\;y^{t+1})}{D_{i}^{t}(x^{t},\;y^{t})}\cdot \frac{D_{i}^{t+1}(x^{t+1},\;y^{t+1})}{D_{i}^{t+1}(x^{t},\;y^{t})}{]}^{1/2}$$

- Malmquist index

Releasing the assumption of constant returns to scale, we further transform the formula above and obtain:

(6) $$TFP_{i,t+1}=\left[\frac{D_{i}^{t+1}{\left(x^{t+1},\;y^{t+1}\right)}_{VRS}}{D_{i}^{t}{\left(x^{t},\;y^{t}\right)}_{VRS}}\right]\times \left[\frac{D_{i}^{t+1}{\left(x^{t+1},\;y^{t+1}\right)}_{CRS}}{D_{i}^{t+1}{\left(x^{t+1},\;y^{t+1}\right)}_{VRS}}\cdot \frac{D_{i}^{t}{\left(x^{t},\;y^{t}\right)}_{VRS}}{D_{i}^{t}{\left(x^{t},\;y^{t}\right)}_{CRS}}\right]\;\times {\left[\frac{D_{i}^{t}{\left(x^{t},\;y^{t}\right)}_{CRS}}{D_{i}^{t+1}{\left(x^{t},\;y^{t}\right)}_{CRS}}\times \frac{D_{i}^{t}{\left(x^{t+1},\;y^{t+1}\right)}_{CRS}}{D_{i}^{t+1}{\left(x^{t+1},\;y^{t+1}\right)}_{CRS}}\right]}^{1/2}$$

In Formula (6), the subscript CRS implies constant returns to scale, and VRS implies variable returns to scale. Then, we decompose the change in TFP from period t to period *t* + 1 into three parts: technical change (TC), pure efficiency change (PE), and scale efficiency change (SE).

(7) $$\{\begin{array}{c} TFP_{i,t+1}=TC_{i,t+1}\times PE_{i,t+1}\times SE_{i,t+1}\\ TC_{i,t+1}={\left[\frac{D_{i}^{t}{\left(x^{t+1},\;y^{t+1}\right)}_{CRS}}{D_{i}^{t+1}{\left(x^{t+1},\;y^{t+1}\right)}_{CRS}}\cdot \frac{D_{i}^{t}{\left(x^{t},\;y^{t}\right)}_{CRS}}{D_{i}^{t+1}{\left(x^{t},\;y^{t}\right)}_{CRS}}\right]}^{1/2}\\ PE_{i,t+1}=\frac{D_{i}^{t+1}{\left(x^{t+1},\;y^{t+1}\right)}_{VRS}}{D_{i}^{t}{\left(x^{t},\;y^{t}\right)}_{VRS}}\\ SE_{i,t+1}=\frac{D_{i}^{t+1}{\left(x^{t+1},\;y^{t+1}\right)}_{CRS}}{D_{i}^{t+1}{\left(x^{t+1},\;y^{t+1}\right)}_{VRS}}\cdot \frac{D_{i}^{t}{\left(x^{t},\;y^{t}\right)}_{VRS}}{D_{i}^{t}{\left(x^{t},\;y^{t}\right)}_{CRS}} \end{array}$$

Overall, the growth of total factor productivity is defined as the product of TC, PE, and SE. We interpret our components as follows: the improvement in technical change results from innovation, and pure efficiency indicates the ability to utilize and allocate resources with a static technical level, while scale efficiency reflects the extent of the achievement of economies of scale.

When the Malmquist indices are greater than 1, it means that TFP rises from period t to period *t* + 1, and the efficiency is improved. When they are equal to 1, it means that TFP remains unchanged, as well as the efficiency. When they are less than 1, it means that TFP and the efficiency decrease from period t to period *t* + 1.

#### 2.1.2. Standard Linear Model

- Technology endogeneity model of new growth theory

Since the middle of the 1980s, new growth theory has developed rapidly; it argues that technology is endogenous, overturning the assumption of the new classic growth theory that technology is exogenous. Since then, a great deal of literature has sprung up in this field. Page [36], Jaffe [37], and Gayle [38] noted that corporate technological advancement is affected by the size of the corporation. Salinas-Jiménez et al. [39] and Battisti et al. [40] connected capital accumulation with productivity growth. Baldwin and Okubo [41], Ottaviano [42], and Combes et al. [43] proposed that corporate production benefits from the development of the regional economy. Accordingly, the initial production function of corporate growth is presented as:

(8) Y=A(SIZE, CLR, RD,t)·F(K, L)

In Formula (8), Y denotes corporate business income; SIZE denotes the corporate size; CLR denotes the capital–labor ratio (i.e., capital accumulation); RD denotes the regional economic development level; t denotes time; A(·) denotes the efficiency function of Hicks-neutral technical progress; and F(·) denotes the combination of K (capital input) and L (labor input).

- The effects of environmental regulation and corporate profitability on technology

The relationship between environmental regulation and technological improvement has been intensively investigated. Hamamoto [44], Cole et al. [45], Zhang et al. [46] and many other scholars drew similar conclusions to the Porter hypothesis. Consequently, we expand the corporate growth model by introducing environmental regulation (denoted by ER) into the efficiency function:

(9) Y=A(SIZE, CLR, RD,ER,t)·F(K, L)

Profit is an important source for corporations to invest in R&D. Fagerberg and Godinho [47] and Cainelli et al. [48] found the key role of profitability in corporate innovations. We thus incorporate corporate profitability (denoted by PRO) into the efficiency function and obtain the model:

(10) Y=A(SIZE, CLR, RD,ER,PRO,t)·F(K, L)

Referring to the practice of Hulten et al. [49], we assume that the efficiency function of Hicks-Neutral A(·) is multivariant, namely:

(11) $$A(SIZE,CLR,RD,ER,PRO,t)=A_{i,0}e^{\lambda_{i}t}SIZE_{i,t}^{\varphi_{i}}CLR_{i,t}^{\gamma_{i}}RD_{i,t}^{\delta_{i}}ER_{i,t}^{\rho_{i}}PRO_{i,t}^{\sigma_{i}}$$

In Formula (11), *i* indicates the corporation; *t* indicates the year; $A_{i,0}$ indicates the initial production efficiency; $\lambda_{i}$ indicates the exogenous technological change; and $\varphi_{i}$, $\gamma_{i}$, $\delta_{i}$, $\rho_{i}$, and $\sigma_{i}$ indicate the influential parameters of corporate technical progress with respect to corporate size, capital–labor ratio, regional economic development level, environmental regulation, and corporate profitability.

By substituting Formula (11) for Formula (10) and dividing both sides by $F\left(K_{i,t},\;L_{i,t}\right)$, we obtain the formula for TFP:

(12) $$TFP_{i,t}=Y_{i,t}/F\left(K_{i,t},\;L_{i,t}\right)=A_{i,0}e^{\lambda_{i}t}SIZE_{i,t}^{\varphi_{i}}CLR_{i,t}^{\gamma_{i}}RD_{i,t}^{\delta_{i}}ER_{i,t}^{\rho_{i}}PRO_{i,t}^{\sigma_{i}}$$

By taking the natural logarithm on both sides of Formula (12), we attain the linear equation:

(13) $$\begin{array}{cc} lnTFP_{i,t}=lnA_{i,0} & +\;\lambda_{i}t+\varphi_{i}lnSIZE_{i,t}+\gamma_{i}lnCLR_{i,t}+\delta_{i}lnRD_{i,t}+\rho_{i}lnER_{i,t}\\ & +\;\sigma_{i}lnPRO_{i,t} \end{array}$$

Ultimately, we postulate that the effect of environmental regulation on corporate TFP is contingent on corporate profitability and vice versa. Facing environmental regulation, corporations with different levels of profits might respond to it distinctly. For example, even with the regulation imposed, corporations may stop technological improvements as long as they are earning profits. In addition, the demand for technological improvement can differ for stringently and laxly regulated corporations given the same level of profits earned. Therefore, we add an interaction term between the two variables into Formula (13):

(14) $$\begin{array}{cc} lnTFP_{i,t}=lnA_{i,0} & +\;\lambda_{i}t+\varphi_{i}lnSIZE_{i,t}+\gamma_{i}lnCLR_{i,t}+\delta_{i}lnRD_{i,t}+\rho_{i}lnER_{i,t}\\ & +\;\sigma_{i}lnPRO_{i,t}+\omega_{i}lnER_{i,t}\times lnPRO_{i,t} \end{array}$$

#### 2.1.3. Spatial Econometric Model

Technology has spillover effects. However, by introducing distance into our analyses, i.e., spatial econometric methods, we can measure the spatial technology spillover that is more precise than the spillover measured by classic econometric approaches.

- Spatial panel econometric model

The general form of the spatial panel econometric model is as follows:

(15) $$\{\begin{array}{c} y_{i,t}=\rho w_{i}^{\prime }y_{t}+x_{i,t}^{\prime }\beta +d_{i}^{\prime }X_{t}\delta +u_{i}+\gamma_{i}+\varepsilon_{i,t}\\ \varepsilon_{i,t}=\varphi m_{i}^{\prime }\varepsilon_{t}+v_{i,t} \end{array}$$

where $y_{i,t}$ denotes the explained variable, and $x_{i,t}^{\prime }$ denotes the explanatory variable. ρ denotes the spatial autocorrelation coefficient; $w_{i}^{\prime }$ denotes the i-th row of the explained variable’s spatial weight matrix W; $d_{i}^{\prime }$ denotes the i-th row of the explanatory variables’ spatial weight matrix D; $m_{i}^{\prime }$ denotes the i-th row of the error term’s spatial weight matrix M; $\rho w_{i}^{\prime }y_{t}$ denotes the spatial lagged term of the explained variable; $d_{i}^{\prime }X_{t}\delta$ denotes the spatial lagged term of the explanatory variable; $u_{i}$ denotes the individual effect of region I; and $\gamma_{i}$ denotes the time effect.

In practice, there are three forms of spatial econometric models that are commonly used: the spatial autoregressive model (SAR), the spatial error model (SEM), and the spatial Durbin model (SDM). SDM simultaneously takes into account the spatial correlations of the explained and explanatory variables. Given this, we adopt SDM to carry out the spatial econometric analyses. When φ=0 in Formula (15), SDM can be obtained, and its theoretical form in this article is shown below:

(16) $$\begin{array}{cc} lnTFP_{i,t}=lnA_{i,0} & +\;\lambda_{i}t+\alpha_{i}lnSIZE_{i,t}+\beta_{i}lnCLR_{i,t}+\gamma_{i}lnRD_{i,t}+\theta_{i}lnER_{i,t}\\ & +\;\mu_{i}lnPRO_{i,t}+\pi_{i}lnER_{i,t}\times lnPRO_{i,t}+\rho_{i}WlnTFP_{i,t}\\ & +\;\sigma_{i}WlnSIZE_{i,t}+\tau_{i}WlnCLR_{i,t}+\varphi_{i}WlnRD_{i,t}+\eta_{i}WlnER_{i,t}\\ & +\;\xi_{i}WlnPRO_{i,t}+\nu_{i}WlnER_{i,t}\times lnPRO_{i,t} \end{array}$$

- Measurement of spatial spillover effects

Because of the utilization of the spatial econometric method, we can easily measure the spatial spillover effects by calculating the average total effect, the average direct effect, and the average indirect effect. The average total effect indicates the average effect of the explanatory variable $x_{i}$ from all regions on the explained variable $y_{i}$. The average direct effect indicates the average effect of the explanatory variable $x_{j,i}$ from region j on the explained variable $y_{i}$. The average indirect effect indicates the average effect of the explanatory variable $x_{i}$ from all other regions on the explained variable $y_{i}$.

A simpler interpretation is that the direct effect of an explanatory variable in region j measures its average effect on the explained variable in region j, while the indirect effect of an explanatory variable in region j measures its average effect on the explained variable in region -j (all regions but j). These interpretations are employed in Section 3 to examine the empirical results and avoid redundant explanations.

### 2.2. Variables and Data

#### 2.2.1. Econometric Models

Based on Formula (14), we place initial production efficiency $A_{i,0}$ and exogenous technological change $\lambda_{i}$ in the random error term and add year dummies $v_{t}$. The form of the normal linear econometric model is as follows:

(17) $$lnTFP_{i,t}=\alpha +\beta_{1}lnER_{i,t}+\beta_{2}lnPRO_{i,t}+\beta_{3}lnSIZE_{i,t}+\beta_{4}lnCLR_{i,t}+\beta_{5}lnRD_{i,t}\;+\beta_{6}lnER_{i,t}\times lnPRO_{i,t}+u_{i}+v_{t}+\varepsilon_{i,t}$$

where $\beta_{i}$s are regression coefficients and are similarly so hereinafter.

Similarly, we can obtain the econometric model of the spatial Durbin model:

(18) $$\begin{array}{cc} lnTFP_{i,t}=\alpha_{0}+\; & \alpha_{1}lnSIZE_{i,t}+\alpha_{2}lnCLR_{i,t}+\alpha_{3}lnRD_{i,t}+\alpha_{4}lnER_{i,t}+\alpha_{5}lnPRO_{i,t}\\ & +\;\alpha_{6}lnER_{i,t}\times lnPRO_{i,t}+\alpha_{7}WlnTFP_{i,t}+\alpha_{8}WlnSIZE_{i,t}\\ & +\;\alpha_{9}WlnCLR_{i,t}+\alpha_{10}WlnRD_{i,t}+\alpha_{11}WlnER_{i,t}\\ & +\;\alpha_{12}WlnPRO_{i,t}+\alpha_{13}WlnER_{i,t}\times lnPRO_{i,t} \end{array}$$

where $\alpha_{i}$s are regression coefficients and are similarly so hereinafter.

To explore how environmental regulation and corporate profitability affect corporate TFP and the mechanism thereof, we need to choose three other variables, TC, PE, and SE, as explained variables to estimate the equations. Consequently, three additional equations are needed:

(19) $$lnTC_{i,t}=\alpha +\beta_{1}lnER_{i,t}+\beta_{2}lnPRO_{i,t}+\beta_{3}lnSIZE_{i,t}+\beta_{4}lnCLR_{i,t}+\beta_{5}lnRD_{i,t}\;+\beta_{6}lnER_{i,t}\times lnPRO_{i,t}+u_{i}+v_{t}+\varepsilon_{i,t}$$

(20) $$lnPE_{i,t}=\alpha +\beta_{1}lnER_{i,t}+\beta_{2}lnPRO_{i,t}+\beta_{3}lnSIZE_{i,t}+\beta_{4}lnCLR_{i,t}+\beta_{5}lnRD_{i,t}\;+\beta_{6}lnER_{i,t}\times lnPRO_{i,t}+u_{i}+v_{t}+\varepsilon_{i,t}$$

(21) $$lnSE_{i,t}=\alpha +\beta_{1}lnER_{i,t}+\beta_{2}lnPRO_{i,t}+\beta_{3}lnSIZE_{i,t}+\beta_{4}lnCLR_{i,t}+\beta_{5}lnRD_{i,t}\;+\beta_{6}lnER_{i,t}\times lnPRO_{i,t}+u_{i}+v_{t}+\varepsilon_{i,t}$$

Likewise, when carrying out the spatial econometric estimations, we also estimate a system of four equations:

(22) $$lnTC_{i,t}=\alpha +\alpha_{1}lnSIZE_{i,t}+\cdots +\alpha_{13}WlnER_{i,t}\times lnPRO_{i,t}$$

(23) $$lnPE_{i,t}=\alpha +\alpha_{1}lnSIZE_{i,t}+\cdots +\alpha_{13}WlnER_{i,t}\times lnPRO_{i,t}$$

(24) $$lnSE_{i,t}=\alpha +\alpha_{1}lnSIZE_{i,t}+\cdots +\alpha_{13}WlnER_{i,t}\times lnPRO_{i,t}$$

#### 2.2.2. Variables Specification

To calculate the TFP of each corporation in the sample from 2013 to 2016, the data of the input element and output element for the period of 2012–2016 are needed. We measure output by business income, capital input by the total assets, and labor input by the number of employees [50,51,52]. 

The existing studies mainly measure environmental regulation by pollution abatement fees or pollution abatement and control expenditures, while this article replaces the conventional environmental regulation variable with the quasi-environmental tax. Referring to the methodology of Bi and Yu [53] and Liu and Gu [25], the quasi-environmental tax in this article is composed of urban construction and maintenance tax, excise, resource tax, farmland occupation tax, and administrative charges that are environment related, many of which are levied at proportional rates based on the production capacity of heavily polluting corporations. The administrative charges include: sewage charges, resource compensation fees, embankment fees, water conservancy construction funds, environmental management funds, environmental protection funds, afforestation fees, land subsidence fees, mining rights fees, water and soil conservation fees, soil erosion compensation fees, mining drainage water resource fee, mineral resource use fee, paid use fee of mineral resources, grassland compensation fee, borax reclamation fee, reclamation fee, mineral resource integration fee, vegetation restoration fee, cultivated land compensation fee, environmental protection fee, sewage treatment fee, sanitation costs, waste disposal fees, coal pipe costs, and waste discharge fees. The unit of this variable is in CNY 10 million.

Corporate profitability in this article is a binary indicator that indicates whether a corporation can earn a positive profit. An unprofitable corporation earns a negative profit and vice versa. Corporate profit (in CNY 10 billion) is measured by the total profit extracted from the corporate annual report. If it is positive, lnPRO is equal to 1, and it equals 0 if the corporation incurs a negative profit. Although profitable corporations can vary widely in their profitability, the value of lnPRO can still be classified with the same value of 1.

With regard to the control variables, the corporate size is computed by taking the natural logarithm of the corporate total assets. The capital–labor ratio is the year-end total assets divided by the year-end number of employees. The regional economic development level is represented by the gross domestic product (GDP, in CNY 1 trillion) of the province in which the corporate address is registered. In terms of the instrumental variable, we use the business income of each firm, denoted by BI (in CNY 10 billion).

#### 2.2.3. Data Sources and Samples

The data sources are as follows: (1) total profit, business income, number of employees, and the total assets data are from the Qianzhan database; (2) the GDP data originate from the China National Bureau of Statistics; (3) the data of the quasi-environmental tax breakdowns are extracted from corporate annual reports by manual sorting by the authors.

The State Council of the People’s Republic of China promulgated the National "12th Five-Year Plan" For Environmental Protection on 15 December 2011, which marked a new round of environmental protection campaigns. To examine how environmental regulation has affected corporations since the campaign commenced, our sample interval starts in 2012. However, the sample interval in this article is from 2013 to 2016 because the method of calculating TFP requires eliminating 2012 from the interval. All the corporations in our sample are China’s heavily polluting listed corporations. The reason why we selected these types of corporations are as follows: first, the environmental information disclosures of the heavily polluting listed corporations are more complete than the other types of corporations, which helps us gain the relevant data on the environmental regulation burden. Second, it is more difficult to collect data on the non-listed corporations. In order to test the possible survivor bias caused by the lack of samples of the non-listed corporations, we counted the number of listed corporations in China’s heavily polluting industries from 2013 to 2016. According to the statistics, among the 27 heavily polluting industries, only four of them have seen a slightly decrease in the number of listed corporations compared with other industries, with increasing number of corporations, as is shown in Table 1, indicating less survivor bias of heavily polluting listed corporations, which may affect Hypothesis 2.

**Figure 1:**
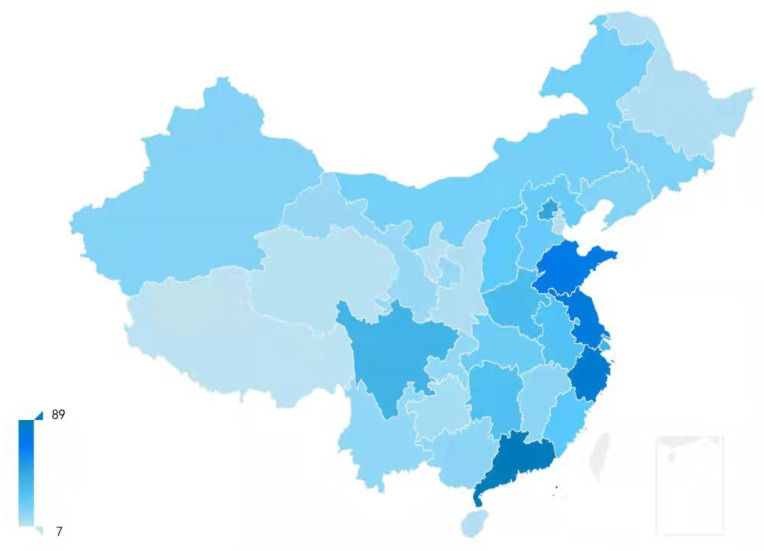
Distribution of sampled heavily polluting listed corporations.

At present, there is no academically specific definition of heavily polluting industries. The criteria of heavily polluting industries in China are stated in The Guide to Environmental Information Disclosures of Listed Corporations (Draft Version) released by the Ministry of Environmental Protection of the People’s Republic of China in 2010, namely the industries of thermal power, steel, cement, electrolytic aluminum, coal, metallurgy, chemicals, petrochemicals, building materials, paper, brewing, pharmaceuticals, fermentation, textile, leather, and mining. Heavily polluting listed corporations are listed corporations classified into heavily polluting industries. In addition, the industry categories of listed corporations are set by the China Securities Regulatory Commission. According to the classification outcome of the industries of listed corporations in Q2 of 2017, we selected 1167 heavily polluting listed corporations. To acquire the relative data of each corporation from 2012 to 2016, we eliminated 252 corporations whose annual reports were published for less than five years, and 15 corporations were also removed from our sample whose data disclosures had faults or missing items. Finally, we obtained a sample of 900 heavily polluting listed corporations, most of which are located in the southeastern coastal area. Figure 1 shows the distribution of the sampled corporations in China (excluding coastal islands). According to the legend, the darker the color of the province, the more corporations there are. Most of the heavily polluting listed corporations are located in the eastern coastal provinces. The reasons are as follows: first, the coastal areas of China are economically developed and populous and so can provide sufficient capital and human resources for the heavily polluting industries. Moreover, the coastal areas are in a high opening degree, which facilitates the transportation of raw materials and products and can therefore attract corporations to operate there. Additionally, the industrial development in the eastern coastal areas of China was relatively early, and the distribution of the heavy industry corporations is intensive there; so, the heavily polluting corporations accounted for a relatively large number.

Environmental regulation mainly affects corporations in the secondary industry, while the primary industry and the tertiary industry are little affected because of their relatively low pollution emissions. In light of the three industry classification provisions released by the National Bureau of Statistics and the classification outcome of the industries of the listed corporations in Q2 of 2017, announced by the China Securities Regulatory Commission, the secondary industry includes 43 industries and 2325 listed corporations. Regardless of the sample interval, our sample contains 27 industries and 1167 listed corporations, which means that the sample covers 62.79% of the industries and 50.19% of the listed corporations that are affected by environmental regulations in China. Thus, the representativeness of our sample is relatively high. We also report summary statistics for the sample in the Table 2.

## 3. Results

### 3.1. Descriptive Statistics

Table 3 reports the statistical features of the variables. As shown in the Table 3, almost all the correlation coefficients between the explained and the explanatory variables are significant at the significance level of 1%, 5%, and 10%, which is the necessary condition for the following regression models, and the correlation coefficients between the explanatory variables are basically lower than 0.8, which means that there is no apparent collinearity in our model.

### 3.2. Results of IV-2SLS Regression

In order to verify Hypothesis 1 and Hypothesis 2, we will use TFP and its Malmquist indexes as explained variables and perform regressions, shown as Equations (17), (19)–(21). In order to make the regression result more robust, we normalized the interaction term $lnER_{i,t}\times lnPRO_{i,t}$ as follows: lnER * lnPRO = (lnER-mlner) * (lnPRO-mlnpro), where mlner and mlnpro are the industry mean of the logarithm of the environmental regulations and the corporations’ profits, respectively.

There might be a bidirectional causality issue between the environmental regulations and the corporate technological levels; so, we adopt fixed-effect IV-2SLS regression to reduce the estimation biases and make causal inferences. We use business income as the instrumental variable for the environmental regulations because business income is highly correlated to the environmental regulation burden as many taxes and fees are based on corporate income, while it is less likely to correlate to the corporate technological levels (and the error term). The Limited Information Maximum Likelihood (LIML) method is also used to test the validity of the instrumental variable. Anderson and Rubin [54,55] introduced the LIML estimator as a way to deal with endogeneity. Many researchers have proven the validity and consistency of the LIML estimation. Wansbeek and Prak [56] suggest that the LIML performs better relative to 2SLS when the instruments are weak, and the LIML appears to have an excellent coverage rate, as it does in the cases where 2SLS is (highly) off the mark. Zhang [57] also thinks that the LIML can effectively solve the influence of the weak exogeneity of the instrumental variables, so that the asymptotic distribution of the estimator on a limited sample maintains the nature of a large sample. Our results indicate that the Cragg–Donald Wald F statistics are much higher than the Stock–Yogo weak ID test critical value at the 5% level, which shows the validity of the instrumental variable. In addition, the results of White’s test and the value of VIF also indicate unrestricted heteroskedasticity and no collinearity. We also control the time effects by adding quadratic time trend terms. The empirical results are shown in Table 4.

The coefficients of lnER in Table 4 show that environmental regulations may have statistically positive effects on the corporations’ efficiency, that is, environmental regulations can increase the TFP, PE, and SE of the sample corporations. The results are consistent with the Porter hypothesis, which proves that environmental regulation can improve the overall efficiency of heavily polluting corporations. However, the coefficient (−0.4029) also indicates a less significant negative effect on technical change, which means that TC perhaps has little or even a negative effect on TFP under environmental regulations. The explanation of this result will be presented in Section 4, Discussion.

Based on the properties of the interaction terms in these models, the results show that for unprofitable corporations, environmental regulations can increase corporate TFP at the significance level of 5%. This effect is weaker for profitable corporations, while corporate profitability may lead to a decrease in corporate TFP, and this impact is amplified by intensifying environmental regulations. With regard to the TFP components, for unprofitable corporations, environmental regulation would decrease TC and increase PE and SE, and the increase in PE is undermined for profitable corporations; the effect on SE is insignificantly different between the two types of corporations. Profitability exerts no impact on TC and SE.

### 3.3. Results of Spatial Econometric Estimations

The spatial spillover effects of the corporations’ technology, which is expressed as Hypothesis 3, will be examined by the regressions shown as Equations (18) and (22)–(24).

The premise of applying spatial econometrics is that there is a spatial correlation among the explained variables. In spatial statistics, we usually utilize Moran’s I [58] index and Geary’s C [59] index to test the existence of spatial correlation. Due to the normality assumption of Moran’s I index, we normalized the variables of TFP and its components TC, PE, SE using Rank cases and then conducted a Shapiro–Wilk normality test. The results are shown in Table 5.

Table 5 reveals that TFP, technical change, pure efficiency change, and scale efficiency change cannot reject the hypothesis of normality distribution. Therefore, the normality assumption for applying Moran’s I index to test the existence of the spatial correlation is satisfied.

Before we embark on calculating the two indices, the spatial weight matrix of sample $W_{ij}$ must be constructed. The value of element $w_{ij}$ equals the reciprocal of the distance between the provincial capital of corporation i’s registered address and that of corporation j. Then, we standardize this matrix so that the sum of all the spatial weights satisfies $\sum_{i=1}^{n}\sum_{j=1}^{n}w_{ij}=n$. The formulae used to calculate the two indices are as follows:

(25) $$I=\frac{\sum_{i=1}^{n}\sum_{j=1}^{n}w_{ij}\left(x_{i}-\bar{x}\right)\left(x_{j}-\bar{x}\right)}{S^{2}\sum_{i=1}^{n}\sum_{j=1}^{n}w_{ij}}$$

(26) $$C=\frac{\left(n-1\right)\sum_{i=1}^{n}\sum_{j=1}^{n}w_{ij}\left(x_{i}-x_{j}\right)}{2\left(\sum_{i=1}^{n}\sum_{j=1}^{n}w_{ij}\right)\left[\sum_{i=1}^{n}{\left(x_{i}-\bar{x}\right)}^{2}\right]}$$

In the formulae above, $S^{2}=\frac{1}{n}\sum_{i=1}^{n}{\left(x_{i}-\bar{x}\right)}^{2}$ is the variance of the sample, and n is the total number of corporations. In general, the range of Moran’s I index is always between −1 and 1. If the value is greater than 0, it means positive autocorrelation; alternatively, it means negative autocorrelation. Geary’s C index generally ranges from 0 to 2, and if the value is greater than 1, it means negative autocorrelation; alternatively, it means positive autocorrelation. The results are shown in Table 6:

Table 6 reveals that TFP, technical change, pure efficiency change, and scale efficiency change have significant positive spatial autocorrelations, which satisfy the premise of adopting the spatial econometric model.

The usual endogeneity considered in classic econometrics is often ignored in spatial econometrics, but an attempt to control for endogeneity can make the estimation less biased and allows us to obtain causality. Our spatial estimation is divided into two stages. In the first stage, we regress the endogenous variables on their instruments and the other exogenous variables to gain the linear predictions of the endogenous variables. In the second stage, we substitute the endogenous variables in the original model with their predicted values in the first stage and run the spatial regression. Note that the spatial Durbin model includes the spatial lagged terms of the explained variables and the explanatory variables, and the impacts of the explanatory variables on the explained variables are not simply reflected by the regressive coefficients. Therefore, we only report the direct, indirect, and total effects and the total effect. The time fixed effect is controlled. The results of the four estimations are shown in Table 7 below.

As is shown in Table 7, the direct effect measures the technology spillover between the heavily polluting corporations in the same province under the environmental regulation. In addition, the indirect effect shows the technology spillovers of the corporations in different provinces. As we assume that the corporations in the same province are all located in the capital city, the distinction between the direct and indirect effects lies in the relative distance between the corporations.

Among the results in Table 7, all the spatial autocorrelated coefficients are positive at the 5% level of significance, illustrating the applicability of the spatial econometric method, which corresponds with the results of Moran’s I indices and Geary’s C in Table 6. Notice that many direct effects are significant while most indirect effects are insignificant. To explain this difference, Tobler’s first law of geography [60] may be a cogent argument, which notes that everything is related to everything else, but things that are closer are more strongly related to each other. 

A notable point to make is that the results of the IV-2SLS regression are nearly consistent with the total effects of the spatial regression. However, the application of spatial econometrics allows us to distinguish the direct effect and the indirect effect that can differ greatly from the results of the standard linear model. We then examine the direct and indirect effects of each variable below.

Environmental regulation may have positive direct effects on TFP. Compared to the unprofitable corporations, this effect is weaker for profitable corporations. It also exerts positive direct effects on PE and SE at the 1% level of significance, although these effects do not differ for different types of corporations and their direct effect on TC is insignificant. Moreover, environmental regulation exhibits no indirect effect on TFP for unprofitable corporations but has a negative indirect effect on TFP for profitable corporations. It also has a positive indirect effect on SE at the 5% level of significance that does not differ for the profitable and unprofitable corporations.

**Figure 2:**
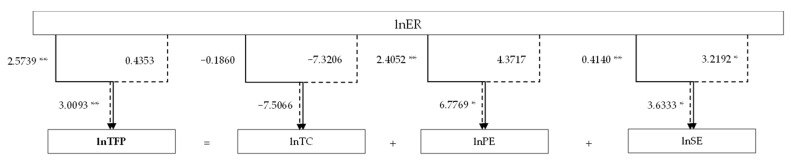
The direct, indirect, and total effects of environmental regulations on corporate efficiency. Note: * and ** indicate significance at the 1% and 5% level.

Figure 2 illustrates the direct, indirect, and total effects of environmental regulation on a corporation’s TFP, TC, PE, and SE. It can be revealed that both the direct and the indirect effects contribute to the total effects. Furthermore, compared with the indirect effects, the direct effects of environmental regulation on corporate efficiency are more significant and have a greater contribution to the total effects. Therefore, we use solid lines and dashed lines to indicate the direct effects and indirect effects, respectively, and the total effects are represented with both of the lines.

Corporate profitability exerts negative direct effects on TFP that may increase as the environmental regulation intensifies. It also has negative direct effects on PE and SE that do not differ between corporations and has no significant direct effect on TC. In terms of the indirect effect, profitability possibly has a negative indirect effect on TFP that increases as the environmental regulation becomes more stringent. It also has a negative indirect effect on PE at the 5% level of significance, which does not differ for the profitable and unprofitable corporations.

Compared with most existing research, this article pays more attention to the research of individual corporations. Through the results interpretation of the spatial Dubin model, it is a concept of relative distance to explore the direct and indirect effects of technology spillover among Chinese corporations under environmental regulation. Furthermore, we further classify corporations according to whether they are profitable or not in order to research the different effects of environmental regulation.

## 4. Discussion

Based on the variable-by-variable results, there are several remarkable points to make.

First, from the perspective of the total effect, it seems that the effects of the explanatory variables are mostly imposed on the pure efficiency change and the scale efficiency change, leading to the change in TFP, while TC is statistically insensitive to all explanatory variables. Basically, it means that, overall, technical change plays no role in affecting TFP in our analyses. However, most of the literature suggests that technical change is positively correlated to TFP [61,62,63]. One justification for this phenomenon is that the corporations in our sample, namely the heavily polluting listed corporations, have no desire or ability to innovate but instead improve their efficiencies. Then, we have to ask whether increases in efficiency changes are sufficient for corporations. The answer lies in the role of technical change. We can run a simple regression to check the contribution of each component of TFP to corporate profitability by controlling environmental regulation and the other control variables. 

To save space, we omit the regression results of environmental regulation and the other control variables. As is shown in the second column of Table 8, compared with the pure efficiency change and the scale efficiency change, the technical change plays a significant role in a corporation’s profit, which indicates a relatively large coefficient. The regression results excluding lnTC (the third column of Table 8) show that the contribution of pure efficiency change to profit and its significance have been reduced, and the coefficient of scale efficiency change has become insignificant. Therefore, technical change plays a significant role in corporations.

Second, environmental regulation may increase the TFP of the unprofitable corporations more than the profitable corporations, and this gap broadens as environmental regulation becomes more stringent. The implication of this phenomenon is that facing an intensifying environmental regulation, unprofitable corporations are forced to increase their technological levels to avoid more losses resulting from taking on more environmental burden. This proposition is based on our observation from the data that the TFP of profitable corporations has a lower mean and variance than unprofitable corporations. This direct effect on TFP is mostly due to efficiency changes. When turning to the indirect effect, environmental regulation exhibits a similar feature that, as it intensifies, the TFP gap between the profitable and unprofitable corporations widens, which suggests that environmental regulation in one province can impact corporate TFP in itself and the other provinces. This indirect effect is also attributed to efficiency changes.

Third, regional economic development contributes to an “outflow” of TFP, i.e., a decrease in the TFP of corporations in the region itself and an increase in corporate TFP in all the other regions, all of which is due to its effect on scale efficiency change; that is, it would increase the scale efficiency in the region per se and decrease the scale efficiency in all the other regions. A possible explanation is that a booming regional economy provides corporations a good opportunity to raise their scale efficiency because the economy of the whole region is in expansion, which may lead to the decrease in scale efficiency for corporations in other regions as their regional economies become comparatively small. Regional economic expansion brings pressure to the management of corporations. For example, it can attract more workers into the region, which makes the pure efficiency of the corporations in this region decrease but contributes to the increase in that of the corporations in the other regions because fewer workers make it easier to manage, which can explain its effect on the TFP of corporations in the local and neighboring regions. However, the changes in TFP resulting from the pure efficiency change are not significant at the 5% level.

## 5. Conclusions

The ongoing debate on the nexus between environmental regulation, corporate economic performance, and technology spillover has attracted many scholars. By decomposing TFP change into technical change, pure efficiency change, and scale efficiency change and applying spatial econometric methods to measure the spatial effects, we find that the direct and indirect effects of environmental regulation and corporate profitability on promoting TFP partly rely on the pure and scale efficiency changes, while the contribution of the predominant component, technical change, is insignificant. As each coin has two sides, environmental regulation acts as a spur to technological improvement but may widen the TFP disparity between profitable and unprofitable corporations. The contribution of this article is mainly reflected in three aspects. Firstly, the quasi-environmental tax is used as the indicator of environmental regulations, which introduces a method of micro-level data measurement for subsequent, related research. Secondly, we proved that environmental regulation would promote the corporations’ TFP and have a more significant effect on unprofitable corporations, which provides guidance for the implementation of environmental regulations. Finally, the research found that the direct effect of spatial spillover is most obvious among the three effects, which reminds policymakers to consider the policy reaction of governments and corporations in the neighboring regions, as well as the countereffects brought by competition.

Based on our findings, we can suggest some policy implications. First, current environmental regulation in China can contribute to the increase in corporate TFP, which provides empirical evidence of an unconflicted relationship between environmental regulation and corporate productivity in China. Thus, in the long run, the existence and improvement of environmental policies can help achieve the improvement of environmental quality and economic growth simultaneously. Second, facing environmental regulation, to increase productivity, corporations in China are inclined to increase their efficiencies instead of technical change, i.e., innovation. For example, many heavily polluting corporations in Beijing have moved to the suburbs of Beijing or even the surrounding provinces because they have little desire to develop and adopt new technologies. Therefore, to enable corporate TFP to increase consistently, governments should design policies that guide or facilitate the corporate innovation that is predominant in the business environment. Third, a provincial environmental regulation can influence the region per se and the other neighboring provinces, which requires the provincial policymakers to adjust their environmental policies according to those from other provinces, instead of regarding their province as an isolated system. Moreover, although environmental regulation incentivizes corporate technological improvement, it also contributes to technological disparities between profitable and unprofitable corporations, implying that the policymaking should strive to reach a balance between the positive and negative sides of environmental regulation.

In the end, we should point out the limitations of this article. When dealing with the endogeneity in our models, we use business income as the instrumental variable for environmental regulation. Even though it passes certain instrument validity tests, it is not the most ideal one and probably brings some biases to our empirical estimations. Additionally, the spatial weight matrix only employs geographical information, ignoring the influences of economic factors. For instance, there is to some extent industrial agglomeration in each province, or the stringencies of environmental regulations vary from province to province. In addition, the value of Moran’s I indices and Geary’s C indices, which are utilized to test the existence of spatial correlation, may be optimized with increasing sample size or observable data. Moreover, taking practicality into consideration when researching spatial spillover at the corporation level, we chose the location of the headquarters as the main activity location of the sample corporations in this article, which can be misleading as the headquarters can be different from the location of the actual activity for tax or prestige reasons. Due to the limitations of this article, some perspectives have not been considered, such as spillover by the industry or pollution type, the influence of the international context on competition and regulatory differences, the inclusion of non-heavily polluting corporations in the research scope, and so on. The above content could be new directions for future research in this field.

## Figures and Tables

**Table 1:** Number of listed corporations in heavily polluting industries (partially).

Industrial Sector	2013	2014	2015	2016
Ferrous Metal Mining and Selection Industry	7	8	8	5
Textile Industry	43	40	39	39
Paper and Paper Products Industry	28	27	26	27
Chemical Fiber Manufacturing Industry	24	24	22	21

**Table 2:** Summary statistics for the sample.

Industrial Sector	Average Scale	Average Employee	Average Business Income	Average Profit	Average Environmental Regulation Burden	Variance of Environmental Regulatory Burden
Coal Mining and Washing Industry (24)	6.26	26,468	14,486.91	426.34	273.82	94,593.30
Oil and Gas Extraction Industry (2)	6.06	4505	1526.53	−271.38	30.98	1653.45
Ferrous Metal Mining and Selection Industry (4)	5.71	4950	3996.89	−837.88	125.55	44,367.00
Non-ferrous Metal Mining and Selection Industry (21)	5.79	5783	11,834.08	438.40	75.63	19,277.02
Mining auxiliary Industry (12)	5.68	9071	9831.87	169.92	24.47	3127.61
Agricultural and Sideline Products Processing Industry (33)	5.42	6220	6483.82	362.55	6.81	319.09
Food Manufacturing Industry (26)	5.51	5445	6164.08	371.60	16.02	727.04
Wine, Beverage, and Refined Tea Manufacturing Industry (34)	5.59	6215	5033.09	1454.17	340.00	522,375.31
Textile Industry (35)	5.43	4925	2738.34	158.48	7.52	65.96
Textile Clothing Industry (22)	5.50	5679	3492.30	383.22	10.02	132.55
Leather, Fur, Feathers, and their Products and Footwear Industry (5)	5.40	4462	1846.28	174.47	6.50	14.79
Wood Processing and Wood, Bamboo, Rattan, Palm, Grass Products Industry (9)	5.41	2277	3029.03	117.43	11.00	363.87
Furniture Manufacturing Industry (5)	5.62	5872	2695.82	375.50	12.12	58.12
Paper and Paper Products Industry (22)	5.69	3881	4211.84	188.49	18.86	678.63
Printing and Recording Media Reproduction Industry (6)	5.34	1934	1268.78	192.85	5.09	19.56
Petroleum Processing, Coking, and Nuclear Fuel Processing Industry (13)	5.74	4714	13,861.08	248.10	974.29	8,096,483.42
Chemical Raw Materials and Chemical Products Manufacturing Industry (155)	5.52	3010	3955.90	183.03	18.12	3653.45
Pharmaceutical Manufacturing Industry (140)	5.51	3623	2924.09	425.32	13.95	327.88
Chemical Fiber Manufacturing Industry (19)	5.66	4111	7122.14	155.76	14.43	1863.72
Rubber and Plastic Products Industry (41)	5.44	3110	3303.99	189.94	7.94	108.40
Non-metallic Mineral Products Industry (64)	5.65	5310	5259.88	494.73	25.30	3071.44
Ferrous Metal Smelting and Calendaring Industry (28)	6.39	16,425	33,096.90	−124.76	63.68	3038.06
Non-ferrous Metal Smelting and Calendaring Industry (54)	5.74	5946	10,745.27	22.56	21.89	2145.45
Metal Products Industry (38)	5.53	4888	5188.08	263.47	10.57	525.01
Electricity, Heat Production and Supply Industry (60)	6.01	3928	8557.99	1481.35	40.95	7745.61
Gas Production and Supply Industry (15)	5.67	3027	4323.32	491.72	9.77	299.62
Water Production and Supply Industry (13)	5.86	2425	2370.15	511.60	5.98	42.50

Note: The numbers in brackets indicate the number of corporations in the given industries. The unit in the table is million RMB.

**Table 3:** Correlated coefficient matrix and statistical features of the variables.

Variable	LnTFP	lnTC	lnPE	lnSE	lnER	lnPRO	lnSIZE	lnCLR	lnRD	lnBI
lnTFP	1									
lnTC	0.0088	1								
lnPE	0.4986 **	−0.5060 **	1							
lnSE	0.0542 **	−0.6841 **	−0.1328 **	1						
lnER	0.0058	0.0316	−0.1315 **	0.1014 **	1					
lnPRO	0.0705 **	0.0046	0.0419 *	−0.0082	0.1006 **	1				
lnSIZE	−0.0302	0.0935 **	−0.2037 **	0.0801 **	0.7217 **	0.0430 **	1			
lnCLR	0.0173	0.1829 **	−0.0335 *	−0.1772 **	0.0259	0.0196	0.3392 **	1		
lnRD	0.0095	0.0376 *	0.0253	−0.0664 **	−0.0693 **	0.0953 **	−0.0494 **	−0.0306	1	
lnBI	0.1078 **	0.0238	−0.1134 **	0.1517 **	0.7199 **	0.0790 **	0.8654 **	0.0983 **	0.0279	1
Mean	−0.0306	−0.8733	0.3101	0.5322	−0.3111	0.8764	1.7250	−1.7531	1.0173	0.7962
SD	0.4378	0.9120	0.7788	0.7512	1.8142	0.3292	0.0924	0.7982	0.7877	1.4074
Min	−8.1117	−3.2189	−5.2983	−6.2146	−16.1181	0	1.3983	−5.1446	−2.5063	−5.4804
Max	6.5312	1.0986	7.1905	4.6636	7.1642	1	2.0136	2.7941	2.0901	5.2454

Note: ** and * indicate significance at the 1% and 5% level.

**Table 4:** Empirical result of the IV-2SLS regression.

Variables	lnTFP	lnTC	lnPE	lnSE
lnER	2.5815 ** (3.81)	−0.4029 * (−2.13)	2.5341 ** (4.30)	0.4506 * (2.57)
lnPRO	−0.4194 * (−2.01)	−0.0406 (−0.55)	−0.2821 (−1.39)	−0.0971 (−1.45)
lnER×lnPRO	−0.3886 * (−2.09)	0.0420 (0.83)	−0.3807 * (−2.23)	−0.0492 (−0.95)
lnSIZE	−30.0526 ** (−3.72)	3.5483 (1.61)	−30.6169 ** (−4.26)	−2.9889 (−1.44)
lnCLR	1.0860 ** (3.36)	−0.0054 (−0.06)	1.0882 ** (3.66)	0.0032 (0.04)
lnRD	−1.3700 (−1.54)	−2.2622 ** (−5.23)	0.0325 (0.04)	0.8592 ** (2.61)
time	−0.3073 * (−2.05)	−1.3579 ** (−17.72)	1.5128 ** (9.94)	−0.4626 ** (−7.21)
time^2^	0.0589 * (2.19)	0.3824 ** (27.28)	−0.3189 ** (−11.57)	−0.0045 (−0.38)
**White’s test**	206.44 **	344.46 **	553.45 **	357.08 **
**VIF**	1.59			
**Cragg–Donald Wald F**	52.103			

Note: z-statistics in parenthesis; robust standard errors; ** and * indicate significance at the 1% and 5% level.

**Table 5:** Results of Shapiro–Wilk normality test for the explained variables.

Variables	2013	2014	2015	2016
TFP	0.132 (−4.996)	0.062 (−6.875)	0.062 (−6.875)	0.062 (−6.875)
TC	1.486 (0.976)	8.108 ** (5.160)	0.135 (−4.938)	0.062 (−6.875)
PE	0.062 (−6.875)	0.069 (−6.576)	0.062 (−6.875)	0.062 (−6.875)
SE	0.062 (−6.875)	0.062 (−6.875)	0.062 (−6.875)	0.062 (−6.875)

Note: t-statistics in parenthesis; robust standard errors; ** indicate significance at the 1% level.

**Table 6:** Moran’s I indices and Geary’s C indices of the explained variables.

Indices	2013	2014	2015	2016
TFP-Moran’s I	0.0590 ** (66.024)	0.0240 ** (16.924)	0.0680 ** (10.238)	0.0450 ** (6.573)
TC-Moran’s I	0.0730 ** (9.111)	0.0690 ** (8.517)	0.0430 ** (5.603)	0.0520 ** (6.486)
PE-Moran’s I	0.0130 ** (9.840)	0.0230 ** (8.948)	0.0640 ** (9.675)	0.0300 ** (8.685)
SE-Moran’s I	0.0650 ** (14.546)	0.0500 ** (6.472)	0.0330 ** (4.733)	0.0490 ** (6.545)
TFP-Geary’s C	0.9320 ** (−5.941)	0.9800 (−1.721)	0.9170 ** (−8.818)	0.9510 ** (−5.348)
TC-Geary’s C	0.9250 ** (−9.176)	0.9300 ** (−8.583)	0.9530 ** (−5.524)	0.9460 ** (−6.585)
PE-Geary’s C	0.9960 (−0.347)	0.9810 (−1.740)	0.9230 ** (−8.187)	0.9690 ** (−2.806)
SE-Geary’s C	0.9240 ** (−7.216)	0.9520 ** (−5.649)	0.9620 ** (−4.169)	0.9460 ** (−6.259)

Notes: z-statistics in parenthesis; ** indicates significance at the 1% level.

**Table 7:** Empirical results of the spatial regression.

Variables	Direct Effect				Indirect Effect				Total Effect			
	lnTFP	lnTC	lnPE	lnSE	lnTFP	lnTC	lnPE	lnSE	lnTFP	lnTC	lnPE	lnSE
lnER	2.5739 ** (17.28)	−0.1860 (−1.04)	2.4052 ** (12.63)	0.4140 ** (2.84)	0.4353 (1.12)	−7.3206 (−1.30)	4.3717 (1.58)	3.2192 * (2.04)	3.0093 ** (6.81)	−7.5066 (−1.30)	6.7769 * (2.35)	3.6333 * (2.21)
lnPRO	−0.2872 ** (−8.36)	0.0882 (1.74)	−0.2744 ** (−4.88)	−0.1115 * (−2.41)	−0.0143 (−0.10)	1.1567 (0.55)	−2.2691 * (−2.32)	1.0660 (1.65)	−0.3015 * (−2.22)	1.2449 (0.58)	−2.5435 * (−2.54)	0.9545 (1.44)
lnER×lnPRO	−0.0903 * (−2.01)	0.0246 (0.74)	−0.1004 (−1.72)	−0.0082 (−0.21)	−0.2202 * (−1.98)	0.7961 (0.61)	−0.6950 (−0.83)	−0.1350 (−0.29)	−0.3105 * (−2.38)	0.8207 (0.61)	−0.7954 (−0.91)	−0.1432 (−0.30)
lnSIZE	−29.6816 ** (−17.34)	1.4250 (0.73)	−28.8992 ** (−12.75)	−2.7679 (1.64)	−5.0010 (−1.16)	83.3778 (1.35)	−47.4880 (−1.51)	−38.0170 * (−2.15)	−34.6826 ** (−6.75)	84.8028 (1.34)	−76.3872 * (−2.32)	−40.7849 (−2.21)
lnCLR	1.0436 ** (15.84)	0.1428 (1.94)	0.9457 ** (11.12)	−0.0419 (−0.78)	0.1712 (0.99)	−0.5284 (−0.22)	0.5419 (0.46)	0.1975 (0.29)	1.2149 ** (6.60)	−0.3855 (−0.16)	1.4876 (1.23)	0.1556 (0.22)
lnRD	−24.7988 ** (−3.46)	−1.3125 (−0.27)	−14.4933 (−1.76)	20.1995 ** (2.73)	23.2171 ** (3.15)	6.8888 (0.92)	12.6124 (1.37)	−25.7617 ** (−3.30)	−1.5817 ** (−4.46)	5.5762 (1.28)	−1.8809 (−0.91)	−5.5622 ** (−4.75)
rho	0.1783 * (2.39)	0.9373 ** (152.30)	0.8314 ** (51.26)	0.7071 ** (31.05)	0.1783 * (2.39)	0.9373 ** (152.30)	0.8314 ** (51.26)	0.7071 ** (31.05)	0.1783 * (2.39)	0.9373 ** (152.30)	0.8314 ** (51.26)	0.7071 ** (31.05)

Note: z–statistics in parenthesis; robust standard errors; ** and * indicate significance at the 1% and 5% level.

**Table 8:** Empirical results of the simple panel regression.

Variables	lnPRO	lnPRO (Excluding lnTC)
lnTC	0.0810 ** (3.56)	-
lnPE	0.0552 ** (3.48)	0.0310 * (2.31)
lnSE	0.0411 * (2.18)	−0.0036 (−0.30)

Note: t-statistics in parenthesis; robust standard errors; ** and * indicate significance at the 1% and 5% level.

## Data Availability

Publicly available datasets were analyzed in this study. This data can be found here: https://www.qianzhan.com/ and http://www.stats.gov.cn/.

## References

[1] (2016). China Environmental Status Bulletin.

[2] Porter M.E. (1991). Toward a Dynamic Theory of Strategy. Strateg. Manag. J..

[3] Berman E., Bui L. (2001). Environmental Regulation and Productivity: Evidence from Oil Refineries. Rev. Econ. Stat..

[4] Iraldo F., Testa F., Frey M. (2009). Is an environmental management system able to influence environmental and competitive performance? The case of the eco-management and audit scheme (EMAS) in the European union. J. Clean. Prod..

[5] Costantini V., Mazzanti M. (2012). On the Green and Innovative Side of Trade Competitiveness? The Impact of Environmental Policies and Innovation on EU exports. Res. Policy.

[6] Li G., Li X., Wang N. (2021). Research on the infuence of environmental regulation on technological innovation efficiency of manufacturing industry in China. Int. J. Environ. Sci. Technol..

[7] Montalvo C.C. (2003). Sustainable production and consumption systems—Cooperation for change: Assessing and simulating the willingness of the firm to adopt/develop cleaner technologies. The case of the In-Bond industry in northern Mexico. J. Clean. Prod..

[8] Ramanathan R., Black A., Nath P., Muyldermans L. (2010). Impact of Environmental Regulations on Innovation and Performance in the UK Industrial Sector. Manag. Decis..

[9] Kneller R., Manderson E. (2012). Environmental Regulations and Innovation activity in UK Manufacturing Industries. Resour. Energy Econ..

[10] Zhang B., Bi J., Yuan Z., Ge J., Liu B., Bu M. (2008). Why do firms engage in environmental management? An empirical study in China. J. Clean. Prod..

[11] Lanoie P., Laurent-Lucchetti J., Johnstone N., Ambec S. (2011). Environmental Policy, Innovation and Performance: New Insights on the Porter Hypothesis. J. Econ. Manag. Strateg..

[12] Sen S. (2015). Corporate Governance, Environmental Regulations and Technological Change. Eur. Econ. Rev..

[13] Rubashkina Y., Galeotti M., Verdolini E. (2015). Environmental Regulation and Competitiveness: Empirical Evidence on the Porter Hypothesis from European Manufacturing Sectors. Energy Policy.

[14] Dechezleprêtre A., Neumayer E., Perkins R. (2015). Environmental regulation and the cross-border diffusion of new technology: Evidence from automobile patents. Res. Policy.

[15] Franco C., Marin G. (2017). The Effect of Within-Sector, Upstream and Downstream Environmental Taxes on Innovation and Productivity. Environ. Resour. Econ..

[16] Rassier D.G., Earnhart D. (2015). Effects of environmental regulation on actual and expected profitability. Ecol. Econ..

[17] Albrizio S., Kozluk T., Zipperer V. (2017). Environmental policies and productivity growth: Evidence across industries and firms. J. Environ. Econ. Manag..

[18] Bitat A. (2018). Environmental regulation and eco-innovation: The Porter hypothesis refined. Eurasian Bus. Rev..

[19] Brunel C., Levinson A. (2016). Measuring the Stringency of Environmental Regulations. Rev. Environ. Econ. Policy.

[20] Botta E., Koźluk T. (2014). Measuring Environmental Policy Stringency in OECD Countries: A Composite Index Approach.

[21] Van Leeuwen G., Mohnen P. (2017). Revisiting the Porter hypothesis: An empirical analysis of Green innovation for the Netherlands. Econ. Innovat. New Technol..

[22] Becker R.A., Pasurka C., Shadbegian R.J. (2013). Do Environmental Regulations Disproportionately Affect Small Businesses? Evidence from the Pollution Abatement Costs and Expenditures Survey. J. Environ. Econ. Manag..

[23] Maddison D. (2006). Environmental Kuznets Curves: A Spatial Econometric Approach. J. Environ. Econ. Manag..

[24] Feng Z., Chen W. (2018). Environmental Regulation, Green Innovation, and Industrial Green Development: An Empirical Analysis Based on the Spatial Durbin Model. Sustainability.

[25] Liu A., Gu X. (2020). Environmental Regulation, Technological Progress and Corporate Profit: Empirical Research Based on the Threshold Panel Regression. Sustainability.

[26] Zhou D., Qiu Y., Wang M. (2021). Does environmental regulation promote enterprise profitability? Evidence from the implementation of China’s newly revised Environmental Protection Law. Econ. Model..

[27] Mbanyele W., Wang F. (2021). Environmental regulation and technological innovation: Evidence from China. Environ. Sci. Pollut. Res..

[28] Nie X., Wu J., Chen Z., Zhang A., Wang H. (2021). Can environmental regulation stimulate the regional Porter effect? Double test from quasi-experiment and dynamic panel data models. J. Clean. Prod..

[29] Wu L., Yang M., Wang C. (2021). Strategic interaction of environmental regulation and its influencing mechanism: Evidence of spatial effects among Chinese cities. J. Clean. Prod..

[30] Peng H., Shen N., Ying H., Wang Q. (2021). Can environmental regulation directly promote green innovation behavior?—Based on situation of industrial agglomeration. J. Clean. Prod..

[31] Liu Y., Liu M., Wang G., Zhao L., An P. (2021). Effect of Environmental Regulation on High-quality Economic Development in China—An Empirical Analysis Based on Dynamic Spatial Durbin Model. Environ. Sci. Pollut. Res..

[32] Kumar S. (2006). Environmentally sensitive productivity growth: A global analysis using Malmquist–Luenberger index. Ecol. Econ..

[33] Adetutu M., Glass A.J., Kenjegalieva K., Sickles R.C. (2015). The effects of efficiency and TFP growth on pollution in Europe: A multistage spatial analysis. J. Prod. Anal..

[34] Fare R., Grosskopf S., Norris M., Zhang Z. (1994). Productivity Growth, Technical Progress, and Efficiency Change in Industrialized Countries. Am. Econ. Rev..

[35] Caves D., Christensen L., Diewart W. (1982). The Economic Theory of Index Numbers and the Measurement of Input, Output, and Productivity. Econom. J. Econom. Soc..

[36] Page J.M. (1984). Firm Size and Technical Efficiency: Applications of Production Frontiers to Indian Survey Data. J. Dev. Econ..

[37] Jaffe A.B. (1988). Demand and Supply Influences in R & D Intensity and Productivity Growth. Rev. Econ. Stat..

[38] Gayle P.G. (2001). Market Concentration and Innovation: New Empirical Evidence on the Schumpeterian Hypothesis.

[39] Salinas-Jiménez M.M., Alvarez-Ayuso I., Delgado-Rodríguez M.J. (2006). Capital accumulation and TFP growth in the EU: A production frontier approach. J. Policy Model..

[40] Battisti M., Del Gatto M., Parmeter C.F. (2018). Labor productivity growth: Disentangling technology and capital accumulation. J. Econ. Growth.

[41] Baldwin R., Okubo T. (2006). Heterogeneous Firms, Agglomeration and Economic Geography: Spatial Selection and Sorting. J. Econ. Geogr..

[42] Ottaviano G.I.P. (2011). ‘New’ New Economic Geography: Firm Heterogeneity and Agglomeration Economies. J. Econ. Geogr..

[43] Combes P.P., Duranton G., Gobillon L., Puga D., Roux S. (2012). The Productivity Advantages of Large Cities: Distinguishing Agglomeration from Firm Selection. Econometrica.

[44] Hamamoto M. (2006). Environmental Regulation and The Productivity of Japanese Industries. Resour. Energy Econ..

[45] Cole M., Elliott R., Okubo T. (2010). Trade, Environmental Regulations and Industrial Mobility: An Industry-Level Study of Japan. Ecol. Econ..

[46] Zhang C., Liu H., Bressers H.T.A., Buchanan K.S. (2011). Productivity Growth and Environmental Regulations—Accounting for Undesirable Outputs: Analysis of China’s Thirty Provincial Regions Using the Malmquist-Luenberger Index. Ecol. Econ..

[47] Fagerberg J., Godinho M.M. (2005). Innovation and Catching-Up. The Oxford Handbook of Innovation.

[48] Cainelli G., Marchi V., De Grandinetti R. (2015). Does the development of environmental innovation require different resources? Evidence from Spanish manufacturing firms. J. Clean. Prod..

[49] Hulten C.R., Bennathan E., Srinivasan S. (2006). Infrastructure, Externalities, and Economic Development: A Study of the India Manufacturing Industry. World Bank Econ. Rev..

[50] Zhang D. (2021). Marketization, environmental regulation, and eco-friendly productivity: A Malmquist–Luenberger index for pollution emissions of large Chinese firms. J. Asian Econ..

[51] Yu D., Li X., Yu J., Li H. (2021). The impact of the spatial agglomeration of foreign direct investment on green total factor productivity of Chinese cities. J. Environ. Manag..

[52] Wang M., Li Y., Liao G. (2021). Research on the Impact of Green Technology Innovation on Energy Total Factor Productivity, Based on Provincial Data of China. Front. Environ. Sci..

[53] Bi Q., Yu L. (2016). Relationship between environmental taxes and enterprise green investment behavior: A panel quantile regression approach. China Popul. Resour. Environ..

[54] Anderson T.W., Rubin H. (1949). Estimation of the parameters of a single equation in a complete system of stochastic equations. Ann. Math. Stat..

[55] Anderson T.W., Rubin H. (1950). The asymptotic properties of estimates of the parameters of a single equation in a complete system of stochastic equations. Ann. Math. Stat..

[56] Wansbeek T., Prak D. (2017). LIML in the static linear panel data model. Econom. Rev..

[57] Zhang X. (2014). Improved method of dynamic panel structure equation estimation based on LIML. Stat. Decis..

[58] Moran P. (1950). Notes on the Continuous Stochastic Phenomena. Biometrika.

[59] Geary R. (1954). The Contiguity Ratio and Statistical Mapping. Inc. Stat..

[60] Tobler W.R. (1970). A Computer Movie Simulating Urban Growth in the Detroit Region. Econ. Geogr..

[61] Managi S., Opaluch J.J., Jin D., Grigalunas T.A. (2005). Environmental Regulations and Technological Change in the Offshore Oil and Gas Industry. Land Econ..

[62] Saal D.S., Parker D., Weyman-Jones T. (2007). Determining the contribution of technological change, efficiency change and scale change to productivity growth in the privatized English and Welsh water and sewerage industry: 1985–2000. J. Prod. Anal..

[63] Galdeano-Gómez E. (2008). Productivity Effects of Environmental Performance: Evidence from TFP Analysis on Marketing Cooperatives. Appl. Econ..

